# Emerging roles of HECT‐type E3 ubiquitin ligases in autophagy regulation

**DOI:** 10.1002/1878-0261.12567

**Published:** 2019-09-05

**Authors:** Gerry Melino, Francesco Cecconi, Pier Giuseppe Pelicci, Tak Wah Mak, Francesca Bernassola

**Affiliations:** ^1^ Department of Experimental Medicine TOR University of Rome “Tor Vergata” Italy; ^2^ Medical Research Council, Toxicology Unit University of Cambridge UK; ^3^ Cell Stress and Survival Unit Danish Cancer Society Research Center Copenhagen Denmark; ^4^ Department of Biology Tor Vergata University of Rome Italy; ^5^ Department of Pediatric Hematology and Oncology IRCCS Bambino Gesù Children's Hospital Rome Italy; ^6^ Department of Experimental Oncology IEO, European Institute of Oncology IRCCS Milan Italy; ^7^ Department of Oncology and Haemato‐Oncology Milan University Italy; ^8^ The Campbell Family Institute for Breast Cancer Research Ontario Cancer Institute Princess Margaret Hospital Toronto ON Canada

**Keywords:** autophagy, HECT E3 ubiquitin ligases, proteasomal degradation, ubiquitylation

## Abstract

Autophagy is a conserved self‐eating process that delivers cytoplasmic material to the lysosome to allow degradation of intracellular components, including soluble, unfolded and aggregated proteins, damaged organelles, and invading microorganisms. Autophagy provides a homeostatic control mechanism and is essential for balancing sources of energy in response to nutrient stress. Autophagic dysfunction or dysregulation has been implicated in several human pathologies, including cancer and neurodegeneration, and its modulation has substantial potential as a therapeutic strategy. Given the relevant clinical and therapeutic implications of autophagy, there is emerging intense interest in the identification of the key factors regulating the components of the autophagic machinery. Various post‐translational modifications, including ubiquitylation, have been implicated in autophagy control. The list of the E3 ubiquitin protein ligases involved in the regulation of several steps of the autophagic process is continuously growing. In this review, we will focus on recent advances in the understanding of the role of the homologous to the E6AP carboxyl terminus‐type E3 ubiquitin ligases in autophagy control.

**Abbreviations**
AMBRA1: activating molecule in BECLIN 1‐regulated autophagy protein 1
AML: acute myeloid leukemia
ATG: autophagy‐related proteins
BH3: Bcl‐2 homology 3 domain
CDL: cullin‐dependent ligase
E1: E1 activating enzyme
E2: E2 conjugating enzyme
E3: E3 ubiquitin ligase
ER: endoplasmic reticulum
HECT: homologous to the E6AP carboxyl terminus
LC3: microtubule‐associated protein light‐chain 3 protein
LIR: LC3‐interacting region
Lm: L. monocytogenes
MFN2: mitofusin 2
Mtb: M. tuberculosis
mTORC1: mammalian target of rapamycin complex 1
NBR1: neighbor of BRCA1 gene
NEDD4: neural precursor cell expressed developmentally down‐regulated protein 4
OPTN: optineurin
PE: phosphatidylethanolamine
PI3P: phosphatidylinositol‐3‐phosphate
PtdIns3K: class‐III phosphatidylinositol 3‐kinase
RDL: regulator of chromosome condensation 1 (RCC1)‐like domain
RING: really interesting new gene
SMURFs: Smad ubiquitin regulatory factors
SPRY: SPRY/B30.2 domain
SQSTM 1: sequestosome 1
TRIM: TRIpartit motif
UBA: ubiquitin‐associated domain
ULK1: Unc‐51‐like kinase 1
UPS: ubiquitin/proteasome system
UVRAG: UV resistance‐associated gene
WD40: W‐D repeat domain
WWE: WWE domain
WWP1: WW domain‐containing E3 ubiquitin protein ligase 1

## 1 The ubiquitin/proteasome system

Ubiquitylation is an ATP‐dependent and reversible enzymatic process that involves the covalent attachment of the conserved polypeptide ubiquitin to target proteins and is highly relevant in a number of physiological and pathological processes (El‐Hachem *et al*., 2018; Ma *et al*., 2018; Sane *et al*., 2018). The outcome of the ubiquitylation reaction is the formation of an isopeptide bond between the ε‐amino group of a Lys residue of the substrate and the C‐terminal Gly76 carboxyl group of ubiquitin. Ubiquitin conjugation to proteins occurs through the sequential and coordinate action of E1 activating (E1), E2 conjugating (E2), and E3 ubiquitin ligase (E3) enzymes that activate, transfer, and ligate ubiquitin to substrates (Ciechanover, 2005). The E3s transfer ubiquitin from the E2 to a substrate or directly promote the attachment of ubiquitin to the target molecule. Given that E3s are the final executioners of ubiquitin tagging, they mainly account for substrate specificity and versatility of the ubiquitylation reaction. Depending on the structural properties of their catalytic structural domains as well as to the mechanism of catalysis, the E3s fall into two major classes, really interesting new gene (RING)‐ and homologous to the E6AP carboxyl terminus (HECT)‐type enzymes. RING‐type E3s contain RING‐finger motifs that serve as scaffolds to bring the E2 in proximity of the substrate and facilitate the transfer of ubiquitin (Ozkan *et al*., 2005). Members of the HECT family of E3s are instead characterized by a highly conserved HECT domain, which directly catalyzes the covalent attachment of ubiquitin to substrate proteins (Rotin and Kumar, 2009).

Proteins can be modified by mono‐ubiquitylation, as a result of the attachment of a single ubiquitin, or by polyubiquitylation through the sequential attachment of ubiquitin molecules on a Lys residue. Ubiquitin has seven lysine residues (K6, K11, K27, K29, K33, K48, and K63) that can become acceptors of another ubiquitin moiety in subsequent rounds of ubiquitylation, eventually leading to the generation of different types of polyubiquitin chains (Xu *et al*., 2009).

Mono‐ or polyubiquitylation and the exact linkage chain composition and topology dictate the distinct fate of the substrates. Recognition and proteolysis of protein substrates by the proteasome are mainly, but not solely, associated with K48‐linked ubiquitin chains. Additionally, K29 and K11 polymers mediate proteasomal degradation, more frequently when found in a mixed or branched chain with K48 and K63 linkages. Substrates conjugated to K63‐linked ubiquitin chains as well as mono‐ubiquitylated proteins are preferentially degraded by the autophagy/lysosome system (Kwon and Ciechanover, 2017).

Chains of at least four ubiquitins mark proteins for transportation and recognition by the proteasome, where proteins are degraded to oligopeptides. These are then released into either the cytoplasm or nucleoplasm, where they are digested into amino acids by soluble peptidases. The proteasome is a large multisubunit organelle, consisting of a central 20S cylinder‐shaped multiprotein complex displaying the proteolytic activity and a 19S regulatory particle at either of its ends. Substrate entry is a complex process which is controlled by the 19S particle. The 19S subunit enables the proteasome to drive binding, deubiquitylation, unfolding, and translocation of target proteins to proteolytic sites. After the substrate enters the 20S's central chamber, polypeptides are broken down by chymotrypsin‐, caspase‐ or trypsin‐like proteolytic sites.

The ubiquitin/proteasome system (UPS) is the major intracellular protein degradation pathway in all eukaryotes. It is responsible for turnover of short‐lived proteins and for the disposal of misfolded proteins. However, ubiquitin does not always signal for protein degradation, but it also controls a number of biological processes such as transcription, enzymatic activation, chromatin remodeling, subcellular relocalization, intracellular trafficking, and DNA repair.

## 2 General features of HECT‐type domain E3s

The HECT E3s (28 known enzymes in human) are endowed with intrinsic enzymatic activity. They employ a catalytic Cys residue located in the HECT domain as an acceptor of ubiquitin from E2s. After loading activated ubiquitin on themselves through the formation of a ubiquitin‐thioester catalytic intermediate, they transfer ubiquitin to a Lys residue in the target protein. HECT E3s recruit substrates through specific protein–protein interaction module within the N‐terminal region, but they can require accessory or adaptor proteins for substrate recognition.

**Figure 1:**
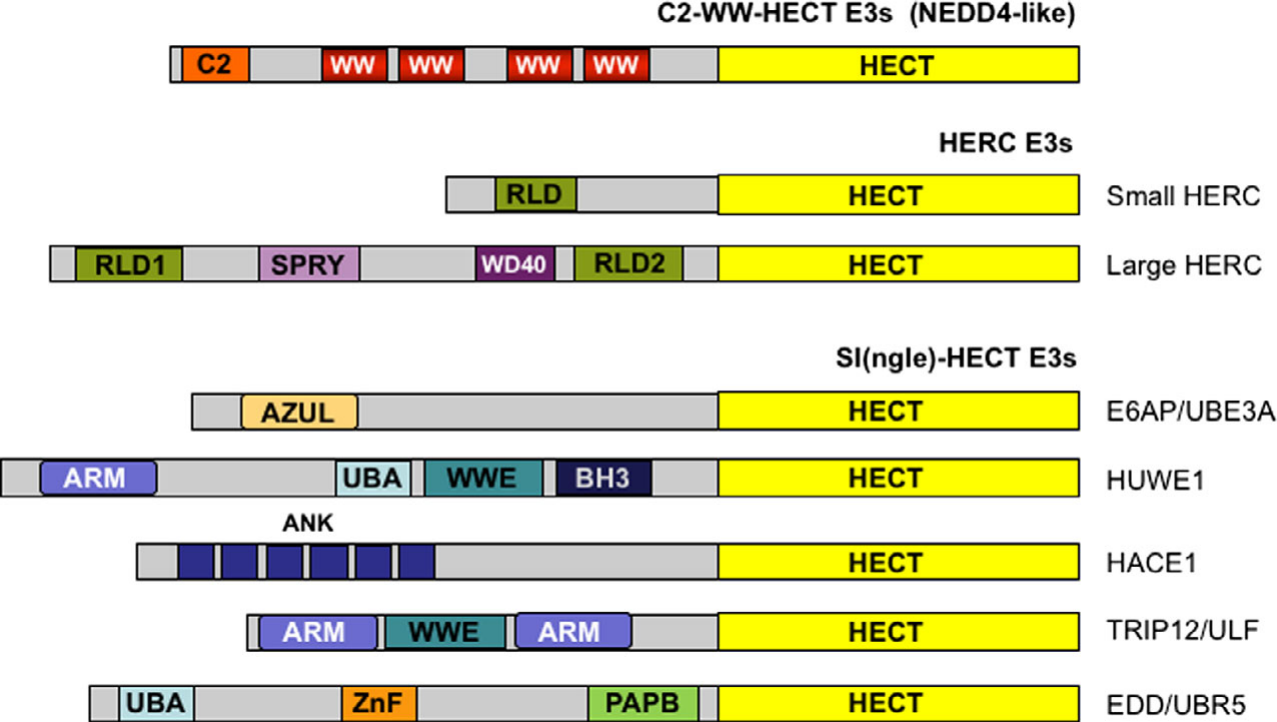
Domain organization of HECT‐type E3 enzymes. The HECT E3s have been assigned to three subgroups according to their N‐terminal protein–protein interaction domains. The C2‐WW‐HECT members are composed of an N‐terminal protein kinase C‐related C2 domain, two‐four central WW domains for substrate recruitment and a C‐terminal HECT domain. HERC E3s have one (small HERC) or more RLDs (large HERC) that precede the HECT domain. Large HERCs contain additional domains, such as SPRY and WD40 domains. The SI(ngle)‐HECT subfamily is characterized by the presence of a HECT domain but lack both WW and RDL domains. These enzymes recruit the substrates through several distinct structural modules including armadillo repeat‐containing domain, amino‐terminal Zn‐finger of Ube3a ligase domain, WWE, BH3 domain, ankyrin repeat‐containing domain, polyadenylate‐binding protein C‐terminal domain, UBA, and zinc finger domain.

On the basis of the distinct structural features of their N‐terminal protein–protein interaction domains, the HECT E3s have been grouped into three subfamilies (Fig. 1). The C2‐WW‐HECT [also designated Neural precursor cell expressed developmentally down‐regulated protein 4 (NEDD4)‐like] subfamily displays a common general modular architecture consisting of an N‐terminal protein kinase C‐related C2 domain, and two to four central tryptophan–tryptophan (WW) protein interacting modules that precede the HECT domain (Fig. 1). This group includes nine members in human: NEDD4‐1, NEDD4‐2, ITCH, Smad ubiquitin regulatory factors (SMURF)1, SMURF2, WW domain‐containing E3 ubiquitin protein ligase 1 (WWP1), WWP2, NEDL1, and NEDL2 (Huibregtse *et al*., 1995; reviewed in Rotin and Kumar, 2009). The C2 domain is a Ca^+2^‐dependent binding domain that mediates the interactions of the C2‐WW‐HECT E3s with phospholipids, inositol phosphate and proteins. As a result, these enzymes are targeted to membrane compartments, including plasma membrane, Golgi apparatus, endosomes, and lysosomes (Angers *et al*., 2004). The WW domains are responsible for the recruitment of protein substrates and adaptors through the recognition of Pro‐rich motifs (preferentially PPxY, and LPxY), and phosphorylated Ser/Thr‐Pro sites.

The HERC subfamily of E3s is characterized by having one or more regulator of chromosome condensation 1 (RCC1)‐like domains (RLDs) for substrate recruitment (Fig. 1). This subgroup can be further classified into the small and the large HERC family members (six enzymes in human). The small HERC E3s (100–120 kDa) contain a single RLD, while the large HERC E3s (> 500 kDa) carry more than one RLDs and additional domains, including SPRY/B30.2 domain (SPRY) and the W‐D repeat domain (WD40).

The HECT family also includes SI(ngle)‐HECT enzymes that contain neither RLDs nor WW domains and recruit substrates through various number and types of protein interacting modules (Fig. 1).

Different HECTs display distinct chain type specificities. They can indeed generate K6, K11, K27, K29, K33, K48, and K63 linkages (Rotin and Kumar, 2009). The activity of HECT E3s can be regulated through the association of their interaction motifs with regulatory proteins that can facilitate substrate recruitment (adaptor and auxiliary proteins) or instead interfere with substrate binding (negative regulators). In addition, intramolecular interactions (e.g., between the C2 and the HECT domains) force the C2‐WW‐HECT E3s in a catalytically inactive state. More generally, the acquisition of an inactive conformation can be achieved through intermolecular associations, oligomerization, or post‐translational modification events (Chen *et al*., 2017; Courivaud *et al*., 2015; Wan *et al*., 2011).

Recent emerging evidence has unveiled a critical connection between HECT‐type domain E3s and the autophagy machinery. The involvement of HECTs in autophagy regulation will be discussed in detail in the next sections.

## 3 Autophagy

Macroautophagy, hereafter termed autophagy, is a highly conserved intracellular catabolic pathway in which cytoplasmic constituents are engulfed by double‐membrane vesicles and subsequently delivered to lysosomes for degradation (Cho *et al*., 2018; Denton *et al*., 2019; Fu *et al*., 2018; Galluzzi *et al*., 2018; Goiran *et al*., 2018; Kim *et al*., 2018; Lindqvist *et al*., 2018). Under homeostatic conditions, basal levels of autophagy are required to remove misfolded or aggregated proteins and damaged organelles, such as mitochondria, endoplasmic reticulum (ER), and peroxisomes. However, autophagic degradation is significantly activated in response to stress conditions to reprogram cell metabolism, meet biosynthetic demands, and allow cell survival. Indeed, autophagy represents an adaptive cellular response to nutrient deprivation, growth factor depletion, infection, oxidative stress hypoxia, and ER stress. In response to these conditions, autophagy preserves the biosynthetic capacity of the cell by supplying amino acids for *de novo* protein synthesis and maintains ATP levels by providing amino acids and free fatty acids for the Krebs cycle. In particular, starvation triggers nonselective autophagy that nonspecifically uptakes any cytoplasmic material. Selective autophagic pathways are instead engaged to target specific potentially harmful cellular components such as aggregated proteins (aggrephagy), damaged organelles (e.g., mitophagy and ERphagy for mitochondria and ER disposal, respectively), or invading microorganisms (xenophagy) for degradation.

**Figure 2:**
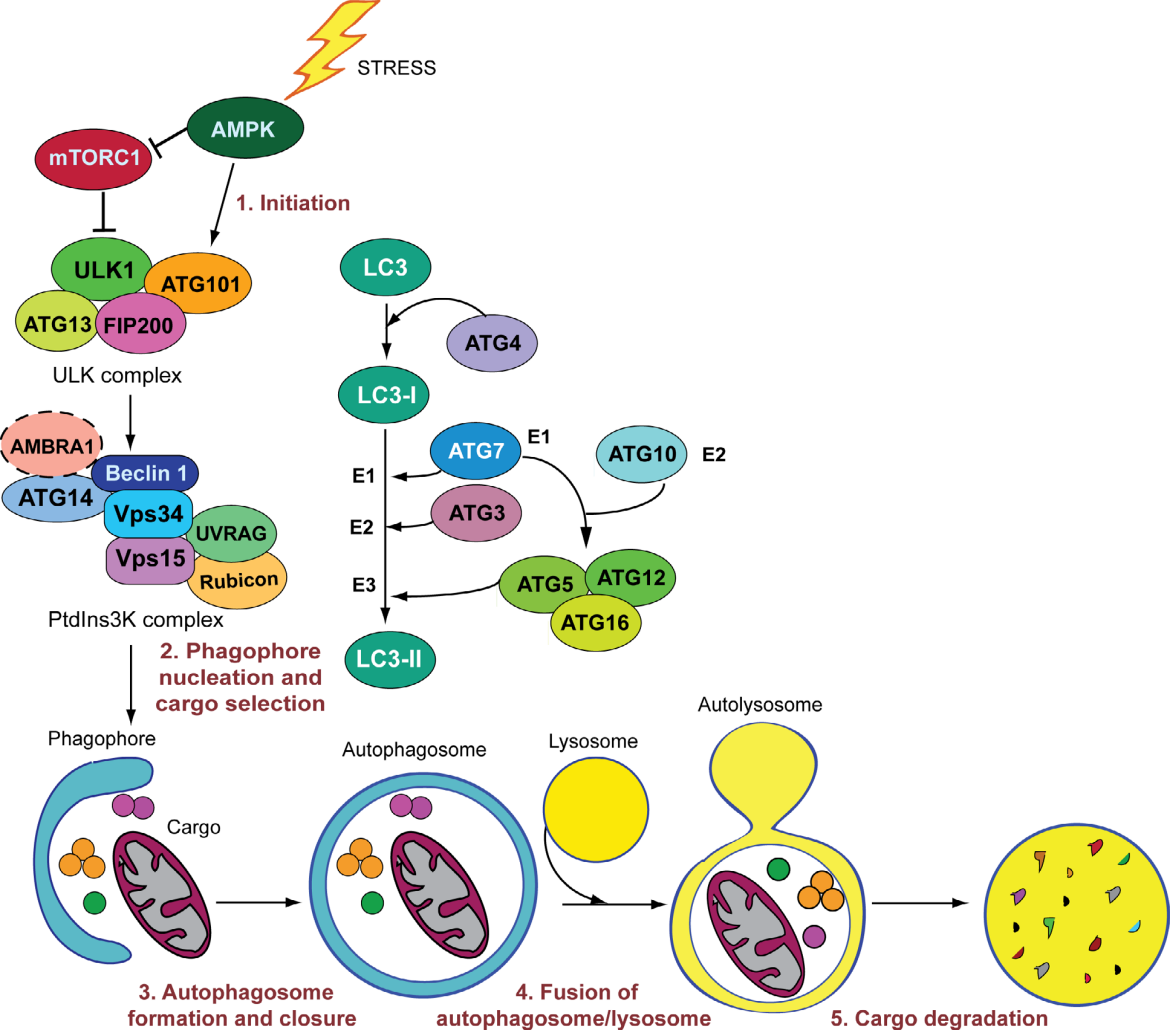
Schematic overview of the autophagy core machinery regulating autophagosome formation. The hierarchy of recruitment, assembly, and activation of ATG proteins during the main phases of the autophagic process are shown. Autophagy is regulated by four ATG protein‐formed complexes, including the ULK1 complex, the PtdIns3K complex, and two ubiquitin‐like protein conjugation systems. The ULK and the PtdIns3K complexes are involved in autophagy initiation and phagophore nucleation, respectively. The ATG4‐processed ATG8s are activated by the E1‐like enzyme ATG7 and then conjugated to membrane‐associated PE, as a result of the coordinated activity of ATG3 and the ATG5‐ATG12‐ATG16L complex, which acts as an E3 ligase. The ATG8 family is essential for biogenesis and elongation of the phagophore and for the recruitment of cargos to the growing phagophore during selective autophagy. A master regulator of the autophagic cascade is mTOR that suppresses PI3P synthesis, indispensable for membrane nucleation, by phosphorylating ULK1, ATG13, and Atg14L.

Autophagy consists of several sequential steps: initiation, phagophore nucleation, autophagosome formation, autophagosome–lysosome fusion, and cargo degradation and recycling (Fig. 2). Induction of autophagy begins with nucleation of an isolation membrane, forming a cup‐shaped structure known as the phagophore, which originates from lipid bilayers contributed mainly by the ER, but also by the Golgi apparatus and endosomes (Abada and Elazar, 2014). A portion of cytoplasm, including organelles, is then sequestered by the elongating phagophore to form an autophagosome, a double‐membrane organelle. Then, the outer membrane of the autophagosome fuses with the lysosomal membrane to form an autolysosome, followed by degradation of the engulfed cytosolic material by the acidic lysosomal hydrolases.

The core autophagic machinery relies on the autophagy‐related (ATG) proteins, which assemble into four functional complexes that are recruited to autophagy membrane compartments and work in sequential order to deliver the cytosolic cargo to the lysosomes (Klionsky *et al*., 2011; Yorimitsu and Klionsky, 2005) (Fig. 2). The upstream regulator of autophagy is the mammalian target of rapamycin complex 1 (mTORC1) (Yang and Klionsky, 2010). Under nutrient‐rich conditions, mTORC1 inhibits autophagy at several steps, such as membrane nucleation, autophagosomal maturation, and recycling of lysosomes from autolysosomes (Kim *et al*., 2011, 2015; Wan *et al*., 2018). Stress conditions that induce the autophagic process (initiation) inhibit mTORC1, causing consequent activation of the serine–threonine kinase Unc‐51‐like kinase 1 complex (ULK1). Stress‐induced autophagy is positively regulated by AMP‐activated protein kinase, which, under conditions of restricted cellular energy, activates the autophagic pathway by phosphorylating ULK1 and by inhibiting mTORC1 (Fig. 2).

Once activated, the ULK1 complex translocates at discrete location sites on the ER, which have been marked by ATG9, and phosphorylates components of the class‐III phosphatidylinositol 3‐kinase (PtdIns3K) complex (Karanasios *et al*., 2016), which, as results, regulate phagophore formation. The PtdIns3K complex comprises BECLIN 1, Vps34/PI3K, Vps15, ATG14L, UV resistance‐associated gene (UVRAG), and Rubicon (Fig. 2). Other proteins, such as activating molecule in BECLIN 1‐regulated autophagy protein 1 (AMBRA1), transiently and less stably associate with this core complex in a condition‐specific manner (Nazio *et al*., 2016). Once phosphorylated, the PtdIns3K complex activates local phosphatidylinositol‐3‐phosphate (PI3P) production at characteristic ER structures called omegasomes. PI3P then recruits the effector proteins, such as DFCP1 and WIPIs, to the omegasome to trigger nucleation of the phagophore. The elongation and expansion of the phagophore membrane are regulated by two ubiquitin‐like conjugation systems (Fig. 2). First, the E1‐like enzyme ATG7 and the E2‐like enzyme ATG10 catalyze the covalent attachment of ATG12 to ATG5 (ATG12~ATG5) (Romanov *et al*., 2012). Then, ATG16L1 forms an E3‐like complex with ATG12~ATG5 (ATG12~ATG5‐ATG16L1). This complex acts in concert with the E1‐like enzyme ATG7 and the E2‐like enzyme ATG3 to drive the lipidation of the ATG8 family members including microtubule‐associated protein light‐chain 3 (LC3) proteins (Hamasaki *et al*., 2013). The conjugation process is preceded by the action of the ATG4 protease, which cleaves the ATG8 proteins to expose the C‐terminal Gly for the subsequent lipidation reaction. Conjugation of membrane‐associated phosphatidylethanolamine (PE) converts LC3 from a freely diffuse into a membrane‐anchored form and is indispensable for phagophore expansion and for facilitating cargo recruitment in selective autophagy. The latter generally requires adaptor proteins, such as p62 [also termed sequestosome 1 (SQSTM 1)] and neighbor of BRCA1 gene (NBR1), which possess a ubiquitin‐associated domain (UBA) and a LC3‐interacting region (LIR) facilitating their binding to LC3. They function as receptors for ubiquitin‐modified cargoes interacting with ubiquitin or polyubiquitin chains via the UBA domain, and then, autophagy adaptors deliver ubiquitylated cargoes to the autophagosome via the LIR domain (Pankiv *et al*., 2007). During mitophagy, a clearance system for removal of damaged mitochondria through the autophagosome–lysosome pathway, recognition of ubiquitin‐modified substrates is mainly mediated by the optineurin (OPTN), NDP52 and AMBRA1 receptors (Di Rita *et al*., 2018; Heo *et al*., 2015; Yamano *et al*., 2016). By acting as bridges between ubiquitin‐tagged mitochondria and LC3, they allow mitochondria delivery to autophagosomes. Similarly, autophagic elimination of intracellular microorganisms requires their targeting with ubiquitin chains, which are then recognized by specific autophagy adaptors, such as p62, NDP52, NBR1, and OPTN, and delivered to autophagosomes (Gomes and Dikic, 2014).

Autophagy controls a variety of physiological and pathophysiological processes including development, aging, neurodegenerative disorders, cancer, and elimination of intracellular pathogens such as viruses, parasites, and bacteria (Levine and Kroemer, 2008).

## 4 Interplay between ubiquitylation and autophagy

The UPS and autophagy were originally regarded as independent degradative pathways with no or few interactions. It is now evident that ubiquitylation regulates multiple steps in autophagy. In addition, the autophagic pathway is activated to compensate for reduced UPS activity, allowing cells to reduce the load of accumulated proteasome‐specific substrates. A link between ubiquitylation and autophagy is established by sharing common regulators or substrates. The most obvious molecule in common between the two pathways is the ubiquitin itself that acts as a signal to mark target substrates for degradation by the proteasome or to be recognized by the adaptor proteins (e.g., p62) to recruit targets into autophagosomes for selective autophagy (Pankiv *et al*., 2007). Regulation of autophagy by protein ubiquitylation is also achieved by affecting the activity, the recruitment as well as the turnover of autophagic components. The existence of cross‐talks among different E3s and deubiquitinases alternate the addition of K48‐ubiquitin chains with K63‐linked polyubiquitylation of constituents of the autophagy core machinery. K63‐linked polyubiquitylation and K48‐linked polyubiquitylation compete each other to activate autophagic proteins in response to stress conditions or to degrade them when the stress situation is resolved, respectively. The contribution of RING‐finger E3s to autophagy has been recently reviewed (Antonioli *et al*., 2017; Cui *et al*., 2016; Grumati and Dikic, 2018). Overall, they have been primarily implicated in the regulation of upstream components of the autophagic machinery, namely mTORC1, the ULK1 and the PtdIns3K complexes. For instance, mTOR is a substrate of the cullin‐dependent ligase complex (CDL) SCF^FBW7^ that negatively regulates its protein stability (Mao *et al*., 2008). Both ULK1 and PtdIns3K complexes are regulated by TRAF6‐mediated ubiquitylation. In particular, K63‐polyubiquitylation promotes stabilization, self‐association, and activation of ULK1 (Nazio *et al*., 2013). Interestingly, here there is a relevant interaction with the major regulator of cell death: BCL‐2 (Adams and Cory, 2018; Montero and Letai, 2018; Pekarsky *et al*., 2018; Pentimalli, 2018; Reinhart *et al*., 2018; Strasser and Vaux, 2018). The attachment of K63 polyubiquitin chains to BECLIN 1 disrupts its inhibitory interaction with BCL‐2, therefore promoting autophagy induction (Shi and Kehrl, 2010). Various members of the TRIpartit motif (TRIM) protein family of E3s have been reported to induce autophagy (reviewed in Antonioli *et al*., 2017). The TRIMs can either participate in the selective recognition of autophagy cargos by cooperating with scaffold protein (e.g., p62), or directly regulate ATGs. Among these E3s, TRIM50 promotes starvation‐induced autophagy, through binding and K63‐polyubiquitylation of BECLIN 1 that facilitates the assembly of BECLIN 1 with ULK1 and, eventually, promotes BECLIN 1 activation (Fusco *et al*., 2018). In response to endomembrane damage affecting phagosomes or lysosomes, TRIM16 associates with and catalyzes K63‐linked polyubiquitylation of ULK1 and BECLIN 1, leading to their stabilization (Chauhan *et al*., 2016). TRIM32 was found to bind ULK1 and AMBRA1 and to stimulate ULK1 phosphorylating activity through AMBRA1‐dependent attachment of K63‐linked polyubiquitin chains (Di Rienzo *et al*., 2019).

On the other hand, K48‐polyubiquitylation‐mediated degradation of autophagy proteins is indispensable to terminate the autophagy response. The main E3s involved in this step are the CDL complexes. Specifically, in unstressed conditions, CDL4 negatively regulates AMBRA1 protein stability (Antonioli *et al*., 2014). Upon nutrient deprivation, ULK1‐dependent Cullin‐4 release determines a transient AMBRA1 stabilization, which is then reverted when the stress becomes prolonged to stop autophagy. In addition, the CDL3 complex catalyzes K48‐linked ubiquitylation of ULK1, BECLIN 1, and VPS34, leading to their degradation (Liu *et al*., 2016).

Among other RING‐finger E3s involved in autophagy inhibition, RNF5 negatively controls the stability of ATG4B (Kuang *et al*., 2012). This Cys protease cleaves the ATG8 family member precursors to expose the C‐terminal Gly that is subsequently conjugated to PE during autophagosome formation. In the next paragraphs, we will highlight the emerging roles of HECT‐type E3s in autophagy regulation (Table 1).

**Table 1:** Protein substrates of the HECT‐type E3s in autophagy regulation

E3	Type of autophagy	Target	Type of poly‐ub chains	References
SMURF1	Xenophagy Starvation‐induced autophagy	Cargo UVRAG	K48‐Ub chains K29, K33‐Ub chains	Orvedahl *et al*. (2011); Feng *et al*. (2019)
SMURF2	Selective autophagy	Cargo (Lamin A)	Unknown	Borroni *et al*. (2018)
NEDD4‐1	Basal and starvation‐induced autophagy Mitophagy Xenophagy	BECLIN 1 p62	K6, K27‐Ub chains K63‐Ub chains	Pei *et al*. (2017); Sun *et al*. (2017); Lin *et al*. (2017)
NEDD4‐2	Basal and ER‐induced autophagy	ULK1	K27, K29 and K63‐Ub chains	Wang *et al*. (2016) Nazio *et al*. (2016)
ITCH	Basal autophagy	Unknown	Unknown	Chhangani *et al*. (2014)
WWP1	Basal autophagy	Unknown	Unknown	Sanarico *et al*. (2018)
HUWE1	Starvation‐induced autophagy Mitophagy	WIPI2 MFN2	Unknown	Wan *et al*. (2018) Di Rita *et al*. (2018)

## 5 HECT‐type E3s in autophagy regulation

### 5.1 SMURFs

Smad ubiquitin regulatory factors SMURF1 and SMURF2 are related members of the NEDD4‐like subfamily. Accessibility of SMURFs toward different substrates is mainly controlled at the level of their subcellular localization. They primarily localize in the nucleus, but continuously shuttle between the nucleus and cytoplasm. Nuclear export arises as a result of binding to adaptor molecules that enrich SMURFs predominantly at the plasma membrane (Kavsak *et al*., 2000). SMURFs also localize to the ER (Guo *et al*., 2011). They are not required for general autophagy, but are regulators of selective autophagy, including xenophagy and mitophagy (Table 1) (Borroni *et al*., 2018; Franco *et al*.,^2017^; Orvedahl *et al*., 2011).

SMURF1 is indispensable for xenophagy‐induced host defense. *Smurf1* ^*−/*−^ murine embryonic fibroblasts are indeed defective in targeting herpes simplex and Sindbis viruses to autophagosomes, suggesting a potential role for SMURF1 in immune defense by targeting viruses to the autophagic machinery (Orvedahl *et al*., 2011). In addition, SMURF1 participates to ubiquitin‐dependent autophagic elimination of intracellular bacteria including M. tuberculosis (Mtb) and L. monocytogenes (Lm). Selective autophagy is achieved by recruiting K48‐linked polyubiquitin chains to bacterial‐associated structures, directing them to LC3 and, subsequently, trafficking Mtb to lysosomes (Franco *et al*., 2017). As a result, SMURF1 restricts bacterial replication in macrophages. Induction of selective autophagy by SMURF1 requires its C2 domain, which is essential for anchoring SMURF1 to membrane phospholipids, specifically to Mtb‐associated structures, and possibly, to the ER. Notably, SMURF1 and the E3 PARKIN work in concert to promote ubiquitin‐dependent selective autophagy of Mtb. The biological relevance of SMURF1‐catalyzed ubiquitylation of Mtb‐associated structures is well exemplified by increased lung bacterial load, pulmonary inflammation, and accelerated mortality that follow the chronic phase of Mtb infection in *Smurf1* ^*−/*−^ mice (Franco *et al*., 2017). Further studies are needed to establish the identity of the substrates targeted for ubiquitylation by SMURF1 and whether it also functions in the autophagic disposal of other intracellular microorganisms.

**Figure 3:**
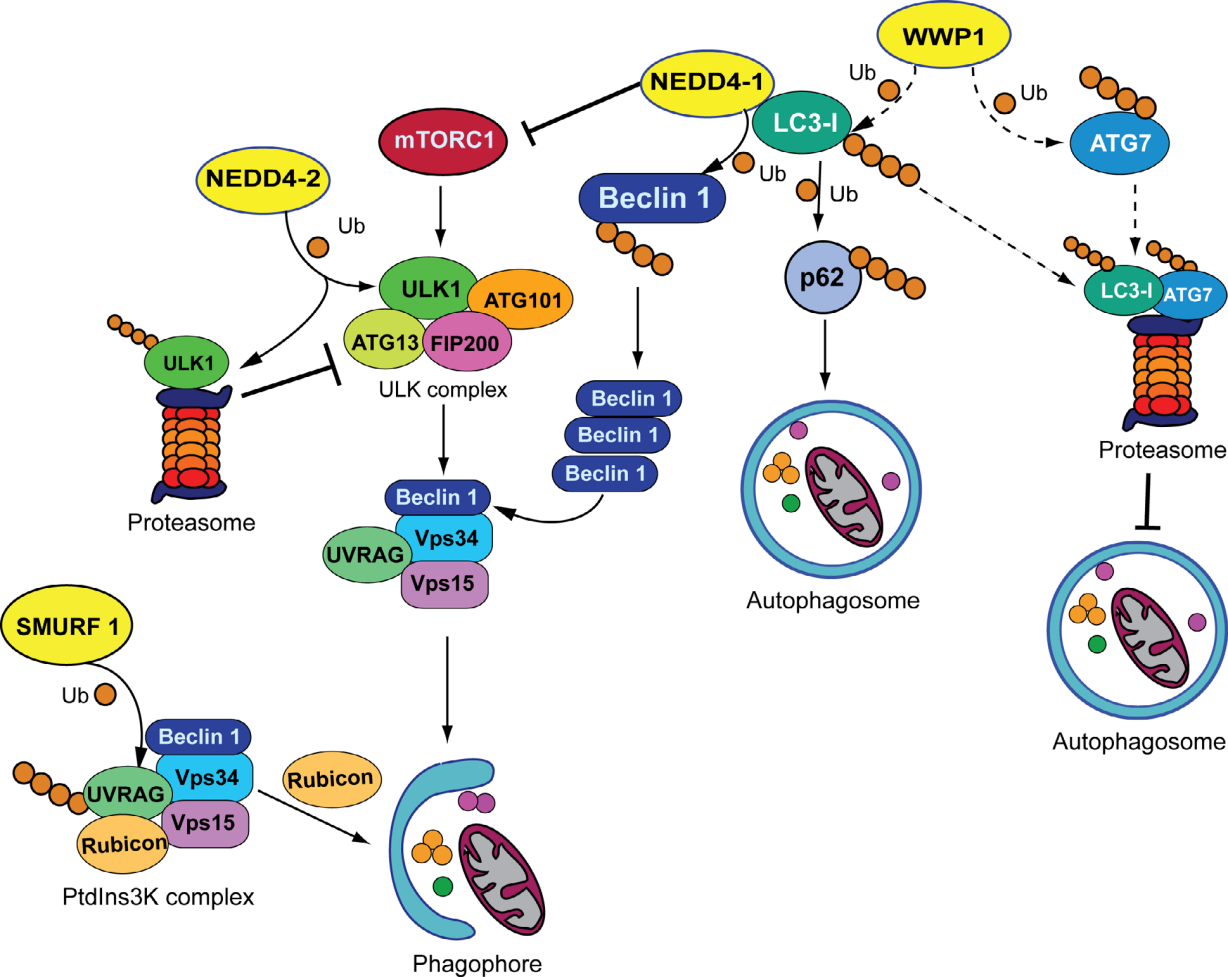
Regulation of the main phases of the autophagic pathway by the C2‐WW‐HECT E3s. Ubiquitin modification of substrates by the C2‐WW‐HECT E3s is mainly nondegradative and affects different steps of the autophagic process: initiation, phagophore nucleation, and autophagosome formation. NEDD4‐1 takes part in the initiation step by negatively modulating the phosphorylation levels of mTOR. In addition, NEDD4‐1 interferes with phagophore formation through K6‐ and K27‐mediated stabilization of BECLIN 1, and with autophagosomal biogenesis by interacting with LC3 and promoting K63‐conjugation of p62. NEDD4‐2 acts as an E3 for ULK1 and regulates its protein stability as an early response to nutrient deprivation. SMURF1 promotes phagophore nucleation by catalyzing K29‐ and K33‐linked UVRAG polyubiquitylation, and subsequent displacement of the inhibitory component Rubicon from the PtdIns3K complex. WWP1 negatively regulates autophagy possibly through the disposal of both LC3 and ATG7.

A recent report by Feng and colleagues has shown that SMURF1 recruits and forms a complex with UVRAG through the PPxY motif (Feng *et al*., 2019). This association results in K29‐ and K33‐linked UVRAG polyubiquitylation, which becomes more pronounced when cells are glucose deprived. This observation is apparently in contrast with all previous findings implying SMURF1 uniquely in selective autophagy (Franco *et al*.,^2017^; Orvedahl *et al*., 2011). Ubiquitylation of UVRAG by SMURF1 impairs its interaction with Rubicon (Fig. 3), which acts as a negative regulator of autophagosome maturation through binding to the PtdIns3K complex. Rubicon interacts with the Vps34/PI3K catalytic subunit thus inhibiting its PI3K lipid kinase activity (Sun *et al*., 2011). Interestingly, ubiquitylation of UVRAG impairs PIK3‐Rubicon interaction, enhances the activity of PIK3, and, eventually, promotes autophagosome maturation.

SMURF2 binds and ubiquitylates Lamin A and its mutant pathogenetic variant progerin (Borroni *et al*., 2018). Lamin filaments provide the nucleus with mechanical stability. Mutations in the gene encoding Lamin A cause genetic premature aging disorders, including Hutchinson–Gilford progeria syndrome (HGPS). Interestingly, SMURF2‐mediated modification of Lamin A promotes its disposal through the autophagic/lysosomal degradation pathway. As a result, SMURF2 negatively affects cellular levels of Lamin A and progerin in primary human dermal fibroblasts derived from healthy individuals and from patients affected by HGPS, respectively. Regulation of progerin by SMURF2 appears to be biologically relevant since ectopic expression of SMURF2 in progeria fibroblasts is able to reduce the nuclear deformability and improve the nuclear circularity, characteristic mechanical features of progeric cells. The clinical implication of this study is that SMURF2‐mediated clearance of progerin represents a potential therapeutic approach in progeria treatment.

### 5.2 NEDD4‐1 and NEDD4‐2

Neural precursor cell expressed developmentally down‐regulated protein 4 is the founding member of the C2‐WW‐HECT subfamily. Along with its closely related homolog NEDD4‐2, NEDD4‐1 plays a crucial role in the regulation of electrolyte homeostasis by controlling the surface abundance of the epithelial Na^+^ channel (Staub *et al*., 1997). Though some controversial findings have been reported on the contribution of NEDD4‐1 to autophagy regulation, the majority of available experimental evidence seems to support a promoting function in basal and starvation‐induced autophagy as well as mitophagy (Table 1) (Lin *et al*., 2017; Pei *et al*., 2017; Sun *et al*., 2017). In addition, similarly to SMURF1, NEDD4‐1 contributes to killing of intracellular Mtb, Lm, and S. pneumoniae during bacterial infection (Pei *et al*., 2017; Ogawa *et al*., 2018). A role in the initiation phase of the autophagic process has been suggested by studies reported by Li and collaborators (Li *et al*., 2015). Silencing of NEDD4‐1 expression impairs starvation‐ and rapamycin‐induced activation of autophagy (Sun *et al*., 2017) and increases the phosphorylation levels of mTOR, suggesting a role for NEDD4‐1 in promoting autophagy activation through the down‐regulation of mTORC1 signaling (Fig. 3). In addition, NEDD4‐1 controls autophagosomal biogenesis by interacting with LC3 through a LIR motif located between the C2 and the WW domains (Pei *et al*., 2017; Sun *et al*., 2017). Interestingly, inactivation of NEDD4‐1 expression impairs autophagosome building and causes the formation of enormous mitochondria, which points out at a severe defect in mitophagy. Mechanistically, LC3 is not a ubiquitylation substrate for NEDD4‐1. However, it has been proposed that the interaction of LC3 with NEDD4‐1 is relevant to recruit NEDD4‐1 to the phagophore and to activate its ligase activity, which ultimately would be required for p62 ubiquitylation (Sun *et al*., 2017) (Fig. 3). NEDD4‐1 polyubiquitylates p62 through K63 conjugation and is indispensable for its function in inclusion body autophagy (Lin *et al*., 2017).

Another component of the autophagic machinery that is ubiquitylated by NEDD4‐1 is BECLIN 1 (Pei *et al*., 2017). NEDD4‐mediated K6‐ and K27‐linked polyubiquitylation of BECLIN 1 results in protein stabilization of BECLIN 1, which, in turn, potentiates autophagy (Fig. 3).

Nazio *et al*. (2016) reported that, in response to nutrient deprivation, ULK1 protein levels are initially destabilized through the activity of NEDD4‐2, while they are restored to basal levels in response to prolonged starvation (Fig. 3). This event is thought to provide a negative control mechanism that limits autophagy overactivation and allows cells to survive prolonged starvation conditions. Furthermore, NEDD4‐2, but not NEDD4‐1, is upregulated in response to ER stress (Wang *et al*., 2016). Perturbations in ER homeostasis cause the accumulation of misfolded proteins and the activation of a signaling network called unfolded protein response. This system ensures the refolding of misfolded polypeptides or their disposal via ubiquitin‐proteasomal degradation and ER stress‐activated autophagy. In particular, in response to ER stress inducers, NEDD4‐2 is upregulated through sXBP1, a transcription factor that regulates the expression of genes necessary to recover ER function. Once induced, NEDD4‐2 modulates ER stress‐induced autophagy, though the mechanism through which this occurs remains to be clarified.

### 5.3 WWP1

WW domain‐containing E3 ubiquitin protein ligase 1 is a well‐established oncogene that has been implicated in the development of several human cancers (Chen *et al*., 2007; Lee *et al*., 2019; Sanarico *et al*., 2018). Unlike the other members of the HECT family, WWP1 functions as a negative regulator of the autophagic pathway (Sanarico *et al*., 2018). WWP1 knockdown in acute myeloid leukemia (AML) cells indeed leads to autophagy induction, which is accompanied by increased total levels of LC3 and ATG7 and accumulation of lipidated LC3. On the contrary, other components of the autophagic machinery seem to be unaltered by WWP1 inactivation. Autophagy activation reduces blast cell survival and contributes to delay leukemia progression in AML cancer xenografts. Although whether or not LC3 and ATG7 might be substrates for WWP1 during autophagy activation is still unexplored, this possibility may represent a conceivable explanation for clarifying how WWP1 interferes with the autophagic machinery. This hypothesis would imply that WWP1 favors degradation of proteins that are involved in the elongation and closure of the autophagosomal membranes to prevent the formation of autophagosomes (Fig. 3). Another observation that supports WWP1 as a regulator of the early steps of autophagy is that the enzyme predominantly localizes to cell membranes (Chen *et al*., 2008), which represent the main nucleation sites for autophagosome formation. Additional studies are required to identify the relevant targets of WWP1 in the context of autophagy regulation.

### 5.4 ITCH

Using a high‐throughput screening approach, Rossi and collaborators have identified clomipramine, an antidepressant drug, as an ITCH inhibitor compound (Rossi *et al*., 2014). Clomipramine efficiently blocks ITCH auto‐ubiquitylation, as well as the modification of its protein substrates. Clomipramine displays some general degree of specificity for other HECT E3s, but it does not inhibit RING‐type E3s. Interestingly, this compound interferes with the autophagic flux blocking the degradation of autophagic cargo (Rossi *et al*., 2009; Rossi *et al.*, 2014). Clomipramine exerts its biological effects at lower micromolar concentrations than those required to inhibit ITCH activity *in vitro*, suggesting that the interference with the autophagic flux may be ITCH‐independent. Nevertheless, these findings arise the intriguing possibility that ITCH may be involved in autophagy regulation to promote the degradation of the serotonin receptor or its recycling mechanism. Clearly, this is an interesting point for future investigations.

Data reported by the group of Chhangani and colleagues seem to corroborate an implication of ITCH in autophagy regulation (Chhangani *et al*., 2014). They provided evidence that ITCH recruits both misfolded and aggregated (e.g., polyglutamine‐containing huntingtin aggregates) cytosolic proteins. They also observed the recruitment of other components of the UPS and of the autophagy machinery to ITCH‐positive protein aggregates. As a result, ITCH promotes the clearance of denatured proteins and expanded‐polyglutamine polypeptides and diminishes protein aggregates. Overall, this study identifies ITCH as a crucial component of the cytoplasmic protein quality control pathway, which provides cytoprotection against misfolded protein‐mediated stress.

### 5.5 HUWE1

HUWE1 (also known as ARF‐BP1, HectH9, URE‐B1, and Mule) is a large E3 (482‐kDa) belonging to the SI(ngle)‐HECT subgroup (Fig. 1). The enzyme contains two N‐terminal ARLDs, an UBA domain, followed by a WWE domain (WWE) and a Bcl‐2 homology 3 (BH3) domain (Fig. 1). HUWE1 catalyzes mono‐ubiquitylation as well as K48‐, K63‐ and K6‐linked ubiquitylation of protein targets. Modification of its substrates crucially regulates cell proliferation, apoptosis, DNA repair, and tumorigenesis (Atsumi *et al*., 2015; Bernassola *et al*., 2008; Chen *et al*., 2005; Zhong *et al*., 2005).

**Figure 4:**
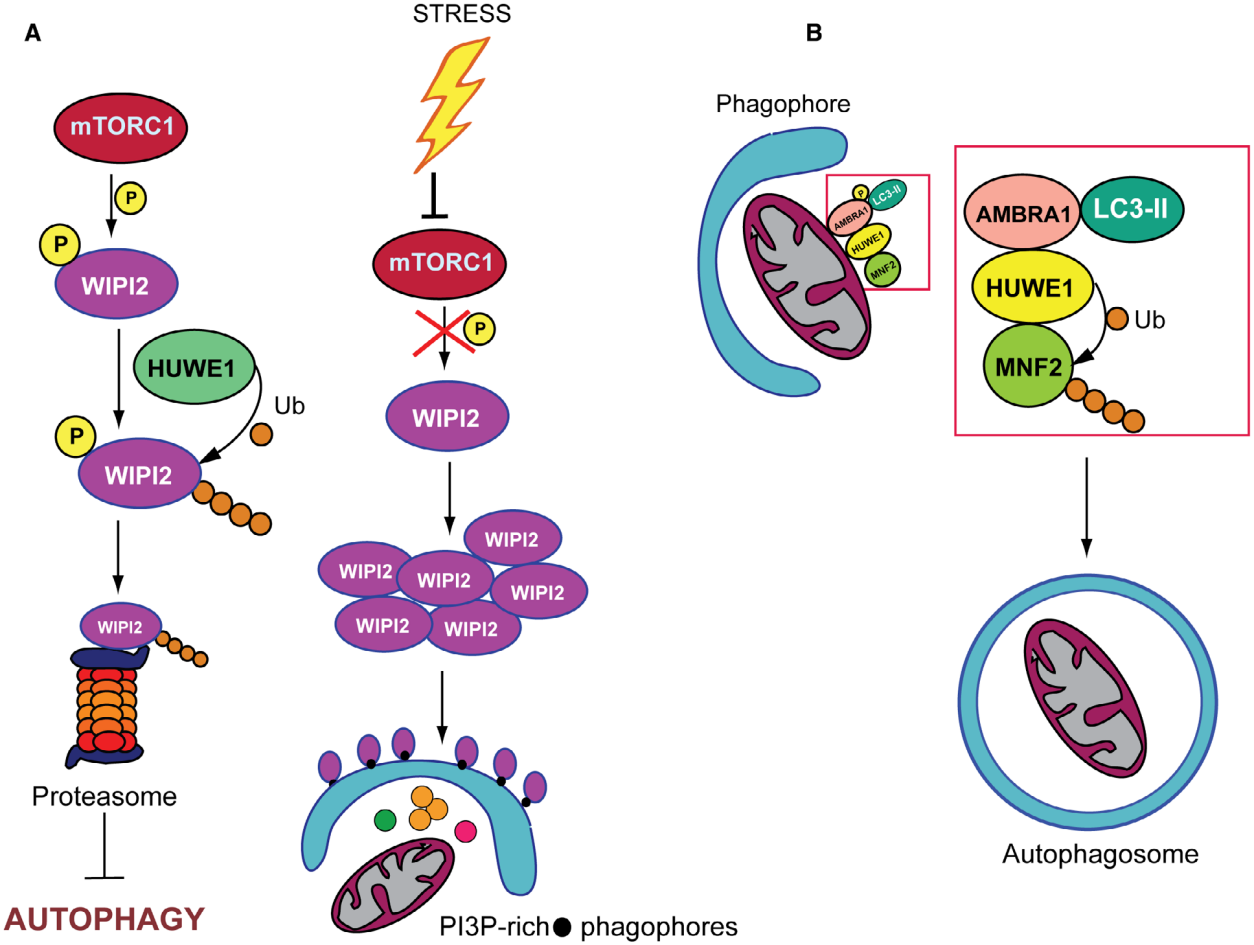
Role of HUWE1 in autophagy regulation. (A) Contribution of HUWE1 to elongation of the phagophore. Under basal conditions, mTORC1‐dependent phosphorylation instructs WIPI2 to interact with HUWE1, resulting in its ubiquitylation and subsequent proteasomal degradation, which, ultimately, prevents autophagy activation. In response to stressors, inhibition of mTORC1 impairs the association of HUWE1 and WIP2. WIP2 then accumulates and recruits the ATG12‐ATG5‐ATG16L complex to the PI3P‐rich phagophores. (B) During mitophagy, AMBRA1 acts as a cofactor for HUWE1 to recruit and ubiquitylates MFN2. Proteasomal degradation of MFN2 facilitates the dissociation of mitochondria from the ER, which is required for mitochondrial degradation.

A report by Wan *et al*. (2018) has recently highlighted WIPI2 as a novel ubiquitylation substrate for HUWE1 (Fig. 4A). During the initial steps of the autophagic process, the recruitment of effector proteins such as WIPI1 and WIPI2 facilitates LC3 lipidation and the subsequent growth and elongation of the phagophore by recruiting the ATG12‐ATG5‐ATG16L complex to the PI3P‐rich phagophores. Binding of WIPI2 by HUWE1 requires mTORC1‐mediated phosphorylation and results in its ubiquitin‐dependent proteasomal degradation, which eventually keeps in check WIPI2 cellular levels and controls the intensity of basal autophagy (Fig. 4A, left). Following autophagy induction, mTORC1 is inactivated and, hence, WIPI2 adopts a dephosphorylated form, which is unable to interact with HUWE1 and therefore undergoes protein stabilization (Fig. 4A, right). WIPI2 is a WD40‐repeat‐containing protein. Some of the HUWE1‐binding proteins contain WD40 domains and are recruited by HUWE1 through this protein–protein interaction module (Thompson *et al*., 2014). Therefore, the authors suggest that mTORC1‐induced phosphorylation of WIPI2 may disrupt the interaction with HUWE1 by interfering with binding mediated by the WD40 domain. An interesting speculation arising from the report by Wan *et al*. is that metabolic adaptation and high mTORC1 activity in cancer cells may have an impact on autophagy regulation, at least in part, through WIPI2 stability control.

HUWE1 has been also implicated in mitophagy regulation (Di Rita *et al*., 2018). During mitophagy, ubiquitylation is a key process that contributes to normal turnover of mitochondrial proteins. PARKIN participates in removing damaged mitochondria by modifying several outer mitochondrial membrane proteins (Heo *et al*., 2015). A central role in selective mitophagy regulation is exerted by AMBRA1 that binds LC3 through a LIR motif, and, acting as a mitophagy receptor, it eventually controls both PARKIN‐dependent and PARKIN‐independent mitochondrial clearance (Strappazzon *et al*., 2015). During PARKIN‐independent mitophagy, AMBRA1 functions as a cofactor for HUWE1 to favor recruitment of HUWE1 to mitochondria and its interaction with mitofusin 2 (MFN2) (Fig. 4B). Cooperation of AMBRA1 with HUWE1 leads to MFN2 ubiquitin‐mediated degradation. Disposal of MFN2 then favors dissociation of mitochondria from the ER, which, ultimately, enhances the rate of mitochondrial degradation (Ashrafi and Schwarz, 2013). AMBRA1‐mediated mitophagy is dependent on IKKα‐induced phosphorylation at Ser1014, flanking its LIR motif. This modification increases binding of AMBRA1 to LC3B and thus fosters its mitophagy receptor function. Notably, HUWE1 potentiates AMBRA1‐activated phosphorylation state, thus establishing a functional crosstalk between HUWE1 and AMBRA1 in mitophagy regulation (Di Rita *et al*., 2018).

## 6 Clinical implications

Deregulation of HECT E3s plays a relevant role in human diseases including neurological disorders (Zhang *et al*., 2011), viral infections (Medina *et al*., 2005), inflammatory diseases (Melino *et al*., 2008), and cancer (Bernassola *et al*., 2008). Emerging evidence has highlighted that HECT E3s play relevant roles in cancer development and progression by regulating the degradation or the activity of both oncogenes and tumor suppressors. As a consequence, abnormal expression, dysfunction, and mutations of the HECT enzymes have been associated with cancer development and chemoresistance (Kao *et al*., 2018; Koganti *et al*., 2018; Lee *et al*., 2019; Melino *et al*., 2008; Sanarico *et al*., 2018).

Similarly, autophagy has been linked to several pathological conditions (Murrow and Debnath, 2013). Paradoxically, autophagy can serve as a protection mechanism but can also contribute to cell damage. The effect of autophagy on cell survival can be variable depending on the cellular context and the intensity and persistence of the stress condition. Autophagy endows cells with an adaptation defense mechanism against cellular stress. This homeostatic function protects against a wide variety of diseases, including neurodegeneration, myopathy, and diabetes. In contrast, the prosurvival functions of autophagy may be deleterious in other disease settings. Autophagy can indeed favor the survival of cancer cells by providing the required cellular intermediates to satisfy their metabolic demands. On the other hand, excessive or prolonged degradation of cellular components ultimately leads to cell death that may or may not display hallmarks of apoptosis (Høyer‐Hansen *et al*., 2005). In this respect, autophagy induction may be beneficial across different pathological conditions including cancer.

Hence, cancer properly exemplifies the dual role of autophagy, which indeed provides a tumor suppressive function during cancer initiation, by removing damaged organelles that may generate free radicals. This eventually limits genomic instability. During cancer progression, autophagy instead favors tumor cell survival within the low‐oxygen and nutrient‐deprived environment and during metastatic dormancy (Lu *et al*., 2008; Smith and Macleod, 2019). In addition, autophagy promotes tumor cell migration and invasion and facilitates the resistance of tumors to radiation and chemotherapy (Kenific *et al*., 2010; Smith and Macleod, 2019).

It becomes evident that HECT E3s might contribute to pathological conditions, at least in part, through autophagy regulation. Modification of UVRAG by SMURF1 was found to be functionally relevant for autophagy‐dependent degradation of the epidermal growth factor receptor and, as a result, for inhibiting hepatocellular carcinoma cell proliferation and tumor growth (Feng *et al*., 2019).

Silencing WWP1 expression in AML blasts activates autophagy which, in turn, decreases viability and induces differentiation of leukemic cells (Sanarico *et al*., 2018). WWP1 overexpression in AML patients indeed causes stabilization of oncoproteins such as PML‐RARα and FLT3/ITD, whose turnover is regulated in an autophagy‐dependent manner. Accumulation of these oncoproteins then accounts for the myeloid maturation block of leukemic cells.

By supporting the high bioenergetic demand of cancer cells, mitophagy sustains tumor growth. HUWE1 may influence cancer metabolism and tumor progression by promoting mitophagy through MFN2 ubiquitylation (Di Rita *et al*., 2018). The role of HUWE1 in tumorigenesis has been very debated. Both tumor suppressive and oncogenic functions have been reported (Kao *et al*., 2018). It has been proposed that the dual role of HUWE1 in cancer is influenced by different protein adaptors or post‐translational modifications. In this respect, AMBRA1 acts as a cofactor for HUWE1 to favor mitophagy induction (Di Rita *et al*., 2018).

## 7 Concluding remarks

Clearly, HECT‐type E3s play a relevant role in the regulation of several steps of the autophagic pathway. With few exceptions (e.g., WWP1, HUWE1), HECT E3s act as autophagy inducers. Current evidence indicates that HECT E3s are mainly involved in the early steps of the autophagy response. Their activities converge toward the regulation of autophagy initiation, nucleation of the phagophore, autophagosome formation, and cargo recruitment in selective autophagy. Autophagy initiation is regulated through the inhibition of mTORC1 by NEDD4‐1, though the underlying molecular mechanism has not been elucidated yet. Phagophore formation regulation is achieved as a result of UVRAG ubiquitylation by SMURF1 that, ultimately, enhances PIK3 activity, and via NEDD4‐1‐mediated modification and stabilization of BECLIN 1. Cargo selection and autophagosome building are regulated by SMURF1 that catalyzes the addition of polyubiquitin chains to bacterial‐associated structures to direct them to LC3. NEDD4‐1 also promotes these steps through p62 ubiquitylation. An example of negative regulation of autophagy by HECT E3s is provided by HUWE1. This enzyme is involved in the ubiquitin‐mediated degradation of the effector protein WIPI2 that is relevant to restrain the intensity of basal autophagy. Furthermore, WWP1 prevents autophagy possibly by interfering with the formation of autophagosomes, though the substrates mediating its function have not been identified yet.

Further exploring the contribution of HECT‐type E3s in autophagy regulation will implement our understanding of the autophagic process. Few inhibitors of the HECT enzymes have been reported to display cytotoxic activities against cancer cells or to increase the response of tumor cells to conventional therapies (Rossi *et al*., 2014; Watt *et al*., 2018; Lee *et al*., 2019). It would be of clinical interest to assess whether these compounds function, at least in part, by interfering with the autophagic pathway. Future studies aimed to identify and characterize HECT E3 inhibitors may lead to the development of novel therapeutic strategies for the treatment of autophagy‐associated pathologies such as cancer and inflammatory disorders.

## Conflict of interest

The authors declare no conflict of interest.

## Author contributions

All authors contributed in writing the manuscript, read, edited, and approved the final manuscript.

## References

[mol212567-bib-0001] Abada A and Elazar Z (2014) Getting ready for building: signaling and autophagosome biogenesis. EMBO Rep 15, 839–852. 25027988 10.15252/embr.201439076 PMC4197041

[mol212567-bib-0002] Adams JM and Cory S (2018) The BCL‐2 arbiters of apoptosis and their growing role as cancer targets. Cell Death Differ 25, 27–36. 29099483 10.1038/cdd.2017.161 PMC5729526

[mol212567-bib-0003] Angers A , Ramjaun AR and McPherson PS (2004) The HECT domain ligase itch ubiquitinates endophilin and localizes to the trans‐Golgi network and endosomal system. J Biol Chem 279, 11471–11479. 14684745 10.1074/jbc.M309934200

[mol212567-bib-0004] Antonioli M , Albiero F , Nazio F , Vescovo T , Perdomo AB , Corazzari M , Marsella C , Piselli P , Gretzmeier C , Dengjel J *et al* (2014) AMBRA1 interplay with cullin E3 ubiquitin ligases regulates autophagy dynamics. Dev Cell 31, 734–746. 25499913 10.1016/j.devcel.2014.11.013

[mol212567-bib-0005] Antonioli M , Di Rienzo M , Piacentini M and Fimia GM (2017) Emerging mechanisms in initiating and terminating autophagy. Trends Biochem Sci 42, 28–41. 27765496 10.1016/j.tibs.2016.09.008

[mol212567-bib-0006] Ashrafi G and Schwarz TL (2013) The pathways of mitophagy for quality control and clearance of mitochondria. Cell Death Differ 20, 31–42. 22743996 10.1038/cdd.2012.81 PMC3524633

[mol212567-bib-0007] Atsumi Y , Minakawa Y , Ono M , Dobashi S , Shinohe K , Shinohara A , Takeda S , Takagi M , Takamatsu N , Nakagama H *et al* (2015) ATM and SIRT6/SNF2H mediate transient H2AX stabilization when DSBs form by blocking HUWE1 to allow efficient gammaH2AX foci formation. Cell Rep 13, 2728–2740. 26711340 10.1016/j.celrep.2015.11.054

[mol212567-bib-0008] Bernassola F , Karin M , Ciechanover A and Melino G (2008) The HECT family of E3 ubiquitin ligases: multiple players in cancer development. Cancer Cell 14, 10–21. 18598940 10.1016/j.ccr.2008.06.001

[mol212567-bib-0009] Borroni AP , Emanuelli A , Shah PA , Ilić N , Apel‐Sarid L , Paolini B , Manikoth Ayyathan D , Koganti P , Levy‐Cohen G and Blank M (2018) Smurf2 regulates stability and the autophagic‐lysosomal turnover of lamin A and its disease‐associated form progerin. Aging Cell 17, e12732. 10.1111/acel.12732 PMC5847874 29405587

[mol212567-bib-0010] Chauhan S , Kumar S , Jain A , Ponpuak M , Mudd MH , Kimura T , Choi SW , Peters R , Mandell M , Bruun JA *et al* (2016) TRIMs and galectins globally cooperate and TRIM16 and galectin‐3 co‐direct autophagy in endomembrane damage homeostasis. Dev Cell 39, 13–27. 27693506 10.1016/j.devcel.2016.08.003 PMC5104201

[mol212567-bib-0011] Chen Z , Jiang H , Xu W , Li X , Dempsey DR , Zhang X , Devreotes P , Wolberger C , Amzel LM , Gabelli SB *et al* (2017) A tunable brake for HECT ubiquitin ligases. Mol Cell 66, 345–357. 28475870 10.1016/j.molcel.2017.03.020 PMC5489419

[mol212567-bib-0012] Chen D , Kon N , Li M , Zhang W , Qin J and Gu W (2005) ARF‐BP1/Mule is a critical mediator of the ARF tumor suppressor. Cell 121, 1071–1083. 15989956 10.1016/j.cell.2005.03.037

[mol212567-bib-0013] Chen C , Zhou Z , Liu R , Li Y , Azmi PB and Seth AK (2008) The WW domain containing E3 ubiquitin protein ligase 1 upregulates ErbB2 and EGFR through RING finger protein 11. Oncogene 27, 6845–6855. 18724389 10.1038/onc.2008.288

[mol212567-bib-0014] Chen C , Zhou Z , Ross JS , Zhou W and Dong JT (2007) The amplified WWP1 gene is a potential molecular target in breast cancer. Int J Cancer 121, 80–87. 17330240 10.1002/ijc.22653

[mol212567-bib-0015] Chhangani D , Upadhyay A , Amanullah A , Joshi V and Mishra A (2014) Ubiquitin ligase ITCH recruitment suppresses the aggregation and cellular toxicity of cytoplasmic misfolded proteins. Sci Rep 4, 5077. 24865853 10.1038/srep05077 PMC4035578

[mol212567-bib-0016] Cho YR , Lee JH , Kim JH , Lee SY , Yoo S , Jung MK , Kim SJ , Yoo HJ , Pack CG , Rho JK *et al* (2018) Matrine suppresses KRAS‐driven pancreatic cancer growth by inhibiting autophagy‐mediated energy metabolism. Mol Oncol 12, 1203–1215. 29791786 10.1002/1878-0261.12324 PMC6026868

[mol212567-bib-0017] Ciechanover A (2005) Intracellular protein degradation: from a vague idea thru the lysosome and the ubiquitin‐proteasome system and onto human diseases and drug targeting. Cell Death Differ 12, 1178–1190. 16094394 10.1038/sj.cdd.4401692

[mol212567-bib-0018] Courivaud T , Ferrand N , Elkhattouti A , Kumar S , Levy L , Ferrigno O and Prunier C (2015) Functional characterization of a WWP1/Tiul1 tumor‐derived mutant reveals a paradigm of its constitutive activation in human cancer. J Biol Chem 290, 21007–21018. 26152726 10.1074/jbc.M115.642314 PMC4543659

[mol212567-bib-0019] Cui D , Xiong X and Zhao Y (2016) Cullin‐RING ligases in regulation of autophagy. Cell Div 11, 8. 27293474 10.1186/s13008-016-0022-5 PMC4902950

[mol212567-bib-0020] Denton D , Xu T , Dayan S , Nicolson S and Kumar S (2019) Dpp regulates autophagy‐dependent midgut removal and signals to block ecdysone production. Cell Death Differ 26, 763–778. 29959404 10.1038/s41418-018-0154-z PMC6460390

[mol212567-bib-0021] Di Rienzo M , Antonioli M , Fusco C , Liu Y , Mari M , Orhon I , Refolo G , Germani F , Corazzari M , Romagnoli A *et al* (2019) Autophagy induction in atrophic muscle cells requires ULK1 activation by TRIM32 through unanchored K63‐linked polyubiquitin chains. Sci Adv 5, eaau8857. 31123703 10.1126/sciadv.aau8857 PMC6527439

[mol212567-bib-0022] Di Rita A , Peschiaroli A , D'Acunzo P , Strobbe D , Hu Z , Gruber J , Nygaard M , Lambrughi M , Melino G , Papaleo E *et al* (2018) HUWE1 E3 ligase promotes PINK1/PARKIN‐independent mitophagy by regulating AMBRA1 activation via IKKα. Nat Commun 9, 3755. 30217973 10.1038/s41467-018-05722-3 PMC6138665

[mol212567-bib-0023] El‐Hachem N , Habel N , Naiken T , Bzioueche H , Cheli Y , Beranger GE , Jaune E , Rouaud F , Nottet N , Reinier F *et al* (2018) Uncovering and deciphering the pro‐invasive role of HACE1 in melanoma cells. Cell Death Differ 25, 2010–2022. 29515254 10.1038/s41418-018-0090-y PMC6219503

[mol212567-bib-0024] Feng X , Jia Y , Zhang Y , Ma F , Zhu Y , Hong X , Zhou Q , He R , Zhang H , Jin J *et al* (2019) Ubiquitination of UVRAG by SMURF1 promotes autophagosome maturation and inhibits hepatocellular carcinoma growth. Autophagy 27, 1–20. 10.1080/15548627.2019.1570063 PMC6613838 30686098

[mol212567-bib-0025] Franco LH , Nair VR , Scharn CR , Xavier RJ , Torrealba JR , Shiloh MU and Levine B (2017) The ubiquitin ligase Smurf1 functions in selective autophagy of *Mycobacterium tuberculosis* and anti‐tuberculous host defense. Cell Host Microbe 21, 59–72. 28017659 10.1016/j.chom.2016.11.002 PMC5699477

[mol212567-bib-0026] Fu S , Wang J , Hu X , Zhou RR , Fu Y , Tang D , Kang R , Huang Y , Sun L , Li N *et al* (2018) Crosstalk between hepatitis B virus X and high‐mobility group box 1 facilitates autophagy in hepatocytes. Mol Oncol 2, 322–338. 10.1002/1878-0261.12165 PMC5830655 29316268

[mol212567-bib-0027] Fusco C , Mandriani B , Di Rienzo M , Micale L , Malerba N , Cocciadiferro D , Sjøttem E , Augello B , Squeo GM , Pellico MT *et al* (2018) TRIM50 regulates Beclin 1 proautophagic activity. Biochim Biophys Acta Mol Cell Res 1865, 908–919. 29604308 10.1016/j.bbamcr.2018.03.011

[mol212567-bib-0028] Galluzzi L , Vitale I , Aaronson SA , Abrams JM , Adam D , Agostinis P , Alnemri ES , Altucci L , Amelio I , Andrews DW *et al* (2018) Molecular mechanisms of cell death: recommendations of the Nomenclature Committee on Cell Death 2018. Cell Death Differ 25, 486–541. 29362479 10.1038/s41418-017-0012-4 PMC5864239

[mol212567-bib-0029] Goiran T , Duplan E , Rouland L , El Manaa W , Lauritzen I , Dunys J , You H , Checler F and Alves da Costa C (2018) Nuclear p53‐mediated repression of autophagy involves PINK1 transcriptional down‐regulation. Cell Death Differ 25, 873–884. 29352272 10.1038/s41418-017-0016-0 PMC5943347

[mol212567-bib-0030] Gomes LC and Dikic I (2014) Autophagy in antimicrobial immunity. Mol Cell 54, 224–233. 24766886 10.1016/j.molcel.2014.03.009

[mol212567-bib-0031] Grumati P and Dikic I (2018) Ubiquitin signaling and autophagy. J Biol Chem 293, 5404–5413. 29187595 10.1074/jbc.TM117.000117 PMC5900779

[mol212567-bib-0032] Guo X , Shen S , Song S , He S , Cui Y , Xing G , Wang J , Yin Y , Fan L , He F *et al* (2011) The E3 ligase Smurf1 regulates Wolfram syndrome protein stability at the endoplasmic reticulum. J Biol Chem 286, 18037–18047. 21454619 10.1074/jbc.M111.225615 PMC3093877

[mol212567-bib-0033] Hamasaki M , Furuta M , Matsuda A , Nezu A , Yamamoto A , Fujita N , Oomori H , Noda T , Haraguchi T , Hiraoka Y *et al* (2013) Autophagosomes form at ER‐ mitochondria contact sites. Nature 495, 389–393. 23455425 10.1038/nature11910

[mol212567-bib-0034] Heo JM , Ordureau A , Paulo JA , Rinehart J and Harper JW (2015) The PINK1‐PARKIN mitochondrial ubiquitylation pathway drives a program of OPTN/NDP52 recruitment and TBK1 activation to promote mitophagy. Mol Cell 60, 7–20. 26365381 10.1016/j.molcel.2015.08.016 PMC4592482

[mol212567-bib-0035] Høyer‐Hansen M , Bastholm L , Mathiasen IS , Elling F and Jäättelä M (2005) Vitamin D analog EB1089 triggers dramatic lysosomal changes and Beclin 1‐mediated autophagic cell death. Cell Death Differ 12, 1297–1309. 15905882 10.1038/sj.cdd.4401651

[mol212567-bib-0036] Huibregtse JM , Scheffner M , Beaudenon S and Howley PM (1995) A family of proteins structurally and functionally related to the E6‐AP ubiquitin‐protein ligase. Proc Natl Acad Sci USA 92, 2563–2567. 7708685 10.1073/pnas.92.7.2563 PMC42258

[mol212567-bib-0037] Kao SH , Wu H and Wu KJ (2018) Ubiquitination by HUWE1 in tumorigenesis and beyond. J Biomed Sci 25, 67. 30176860 10.1186/s12929-018-0470-0 PMC6122628

[mol212567-bib-0038] Karanasios E , Walker SA , Okkenhaug H , Manifava M , Hummel E , Zimmermann H , Ahmed Q , Domart MC , Collinson L and Ktistakis NT (2016) Autophagy initiation by ULK complex assembly on ER tubulovesicular regions marked by ATG9 vesicles. Nat Commun 7, 12420. 27510922 10.1038/ncomms12420 PMC4987534

[mol212567-bib-0039] Kavsak P , Rasmussen RK , Causing CG , Bonni S , Zhu H , Thomsen GH and Wrana JL (2000) Smad7 binds to Smurf2 to form an E3 ubiquitin ligase that targets the TGF beta receptor for degradation. Mol Cell 6, 1365–1375. 11163210 10.1016/s1097-2765(00)00134-9

[mol212567-bib-0040] Kenific CM , Thorburn A and Debnath J (2010) Autophagy and metastasis: another double‐edged sword. Curr Op Cell Biol 22, 1–5. 19945838 10.1016/j.ceb.2009.10.008 PMC2854304

[mol212567-bib-0041] Kim JH , Choi TG , Park S , Yun HR , Nguyen NNY , Jo YH , Jang M , Kim J , Kim J , Kang I *et al* (2018) Mitochondrial ROS‐derived PTEN oxidation activates PI3K pathway for mTOR‐induced myogenic autophagy. Cell Death Differ 25, 1921–1937. 30042494 10.1038/s41418-018-0165-9 PMC6219511

[mol212567-bib-0042] Kim YM , Jung CH , Seo M , Kim EK , Park JM , Bae SS and Kim DH (2015) mTORC1 phosphorylates UVRAG to negatively regulate autophagosome and endosome maturation. Mol Cell 57, 207–218. 25533187 10.1016/j.molcel.2014.11.013 PMC4304967

[mol212567-bib-0043] Kim J , Kundu M , Viollet B and Guan KL (2011) AMPK and mTOR regulate autophagy through direct phosphorylation of Ulk1. Nat Cell Biol 13, 132–141. 21258367 10.1038/ncb2152 PMC3987946

[mol212567-bib-0044] Klionsky DJ , Baehrecke EH , Brumell JH , Chu CT , Codogno O , Cuervo AM , Dednath J , Deretic V , Elazar Z , Eskelinen EL *et al* (2011) A comprehensive glossary of autophagy‐ related molecules and processes. Autophagy 7, 1273–1294. 21997368 10.4161/auto.7.11.17661 PMC3359482

[mol212567-bib-0045] Koganti P , Levy‐Cohen G and Blank M (2018) Smurfs in protein homeostasis, signaling, and cancer. Front Oncol 8, 295. 30116722 10.3389/fonc.2018.00295 PMC6082930

[mol212567-bib-0046] Kuang E , Okumura CY , Sheffy-Levin S , Varsano T , Shu VC , Qi J , Niesman IR , Yang HJ , López-Otín C , Yang WY *et al* (2012) Regulation of ATG4B stability by RNF5 limits basal levels of autophagy and influences susceptibility to bacterial infection. PLoS Genet. 8, e1003007. 23093945 10.1371/journal.pgen.1003007 PMC3475677

[mol212567-bib-0047] Kwon YT and Ciechanover A (2017) The ubiquitin code in the ubiquitin‐proteasome system and autophagy. Trends Biochem Sci 42, 873–886. 28947091 10.1016/j.tibs.2017.09.002

[mol212567-bib-0048] Lee YR , Chen M , Lee JD , Zhang J , Lin SY , Fu TM , Chen H , Ishikawa T , Chiang SY , Katon J *et al* (2019) Reactivation of PTEN tumor suppressor for cancer treatment through inhibition of a MYC‐WWP1 inhibitory pathway. Science 364, eaau0159. 31097636 10.1126/science.aau0159 PMC7081834

[mol212567-bib-0049] Levine B and Kroemer G (2008) Autophagy in the pathogenesis of disease. Cell 132, 27–42. 18191218 10.1016/j.cell.2007.12.018 PMC2696814

[mol212567-bib-0050] Li Y , Zhang L , Zhou J , Luo S , Huang R , Zhao C and Diao A (2015) Nedd4 E3 ubiquitin ligase promotes cell proliferation and autophagy. Cell Prolif 48, 338–347. 25809873 10.1111/cpr.12184 PMC6495840

[mol212567-bib-0051] Lin Q , Dai Q , Meng H , Sun A , Wei J , Peng K , Childress C , Chen M , Shao G and Yang W (2017) The HECT E3 ubiquitin ligase NEDD4 interacts with and ubiquitylates SQSTM1 for inclusion body autophagy. J Cell Sci 130, 3839–3850. 29021346 10.1242/jcs.207068

[mol212567-bib-0052] Lindqvist LM , Frank D , McArthur K , Dite TA , Lazarou M , Oakhill JS , Kile BT and Vaux DL (2018) Autophagy induced during apoptosis degrades mitochondria and inhibits type I interferon secretion. Cell Death Differ 25, 782–794. 10.1038/s41418-017-0017-z PMC5864185 29229994

[mol212567-bib-0053] Liu CC , Lin YC , Chen YH , Chen CM , Pang LY , Chen HA , Wu PR , Lin MY , Jiang ST , Tsai TF *et al* (2016) Cul3–KLHL20 ubiquitin ligase governs the turnover of ULK1 and VPS34 complexes to control autophagy termination. Mol Cell 61, 84–97. 26687681 10.1016/j.molcel.2015.11.001

[mol212567-bib-0054] Lu Z , Luo RZ , Lu Y , Zhang X , Yu Q , Khare S , Kondo S , Kondo Y , Yu Y , Mills GB *et al* (2008) The tumor suppressor gene ARHI regulates autophagy and tumor dormancy in human ovarian cells. J Clin Invest 118, 3917–3929. 19033662 10.1172/JCI35512 PMC2582930

[mol212567-bib-0055] Ma J , Lu Y , Zhang S , Li Y , Huang J , Yin Z , Ren J , Huang K , Liu L , Yang K *et al* (2018) β‐Trcp ubiquitin ligase and RSK2 kinase‐mediated degradation of FOXN2 promotes tumorigenesis and radioresistance in lung cancer. Cell Death Differ 25, 1473–1485. 29396548 10.1038/s41418-017-0055-6 PMC6113220

[mol212567-bib-0056] Mao JH , Kim IJ , Wu D , Climent J , Kang HC and DelRosario R (2008) FBXW7 targets mTOR for degradation and cooperates with PTEN in tumor suppression. Science 321, 1499–5102. 18787170 10.1126/science.1162981 PMC2849753

[mol212567-bib-0057] Medina G , Zhang Y , Tang Y , Gottwein E , Vana ML , Bouamr F , Leis J and Carter CA (2005) The functionally exchangeable L domains in RSV and HIV‐1 Gag direct particle release through pathways linked by Tsg101. Traffic 6, 880–894. 16138902 10.1111/j.1600-0854.2005.00323.x PMC2692930

[mol212567-bib-0058] Melino G , Gallagher E , Aqeilan RI , Knight R , Peschiaroli A , Rossi M , Scialpi F , Malatesta M , Zocchi L , Browne G *et al* (2008) Itch: a HECT‐type E3 ligase regulating immunity, skin and cancer. Cell Death Differ 15, 1103–1112. 18552861 10.1038/cdd.2008.60

[mol212567-bib-0059] Montero J and Letai A (2018) Why do BCL‐2 inhibitors work and where should we use them in the clinic? Cell Death Differ 25, 56–64. 29077093 10.1038/cdd.2017.183 PMC5729538

[mol212567-bib-0060] Murrow L and Debnath J (2013) Autophagy as a stress‐response and quality‐control mechanism: implications for cell injury and human disease. Annu Rev Pathol 8, 105–137. 23072311 10.1146/annurev-pathol-020712-163918 PMC3971121

[mol212567-bib-0061] Nazio F , Carinci M , Valacca C , Bielli P , Strappazzon F , Antonioli M , Ciccosanti F , Rodolfo C , Campello S , Fimia GM *et al* (2016) Fine‐tuning of ULK1 mRNA and protein levels is required for autophagy oscillation. J Cell Biol 215, 841–856. 27932573 10.1083/jcb.201605089 PMC5166502

[mol212567-bib-0062] Nazio F , Strappazzon F , Antonioli M , Bielli P , Cianfanelli V , Bordi M , Gretzmeier C , Dengjel J , Piacentini M , Fimia GM *et al* (2013) mTOR inhibits autophagy by controlling ULK1 ubiquitylation, self‐association and function through AMBRA1 and TRAF6. Nat Cell Biol 15, 406–416. 23524951 10.1038/ncb2708

[mol212567-bib-0063] Ogawa M , Matsuda R , Takada N , Tomokiyo M , Yamamoto S , Shizukusihi S , Yamaji T , Yoshikawa Y , Yoshida M , Tanida I *et al* (2018) Molecular mechanisms of Streptococcus pneumoniae‐targeted autophagy via pneumolysin, Golgi‐resident Rab41, and Nedd4‐1‐mediated K63‐linked ubiquitination. Cell Microbiol 20, e12846. 29582580 10.1111/cmi.12846

[mol212567-bib-0064] Orvedahl A , Sumpter R Jr , Xiao G , Ng A , Zou Z , Tang Y , Narimatsu M , Gilpin C , Sun Q , Roth M *et al* (2011) Image‐based genome‐wide siRNA screen identifies selective autophagy factors. Nature 480, 113–117. 22020285 10.1038/nature10546 PMC3229641

[mol212567-bib-0065] Ozkan E , Yu H and Deisenhofer J (2005) Mechanistic insight into the allosteric activation of a ubiquitin‐conjugating enzyme by RING‐type ubiquitin ligases. Proc Natl Acad Sci USA 102, 18890–18895. 16365295 10.1073/pnas.0509418102 PMC1316884

[mol212567-bib-0066] Pankiv S , Clausen TH , Lamark T , Brech A , Bruun JA , Outzen H , Øvervatn A , Bjørkøy G and Johansen T (2007) P62/SQSTM1 binds directly to Atg8/LC3 to facilitate degradation of ubiquitinated protein aggregates by autophagy. J Biol Chem 282, 24131–24145. 17580304 10.1074/jbc.M702824200

[mol212567-bib-0067] Pei G , Buijze H , Liu H , Moura‐Alves P , Goosmann C , Brinkmann V , Kawabe H , Dorhoi A and Kaufmann SHE (2017) The E3 ubiquitin ligase NEDD4 enhances killing of membrane‐perturbing intracellular bacteria by promoting autophagy. Autophagy 13, 2041–2055. 29251248 10.1080/15548627.2017.1376160 PMC5788543

[mol212567-bib-0068] Pekarsky Y , Balatti V and Croce CM (2018) BCL2 and miR‐15/16: from gene discovery to treatment. Cell Death Differ 25, 21–26. 28984869 10.1038/cdd.2017.159 PMC5729525

[mol212567-bib-0069] Pentimalli F (2018) BCL2: a 30‐year tale of life, death and much more to come. Cell Death Differ 25, 7–9. 29125600 10.1038/cdd.2017.189 PMC5729543

[mol212567-bib-0071] Reinhart R , Rohner L , Wicki S , Fux M and Kaufmann T (2018) BH3 mimetics efficiently induce apoptosis in mouse basophils and mast cells. Cell Death Differ 25, 204–216. 28960207 10.1038/cdd.2017.154 PMC5729523

[mol212567-bib-0072] Romanov J , Walczak M , Ibiricu I , Schuchner S , Ogris E , Kraft C and Martens S (2012) Mechanism and functions of membrane binding by the Atg5–Atg12/Atg16 complex during autophagosome formation. EMBO J 31, 4304–4317. 23064152 10.1038/emboj.2012.278 PMC3501226

[mol212567-bib-0073] Rossi M , Munarriz ER , Bartesaghi S , Milanese M , Dinsdale D , Guerra‐Martin MA , Bampton ET , Glynn P , Bonanno G , Knight RA *et al* (2009) Desmethylclomipramine induces the accumulation of autophagy markers by blocking autophagic flux. J Cell Sci 122, 3330–3339. 19706685 10.1242/jcs.048181 PMC2736865

[mol212567-bib-0074] Rossi M , Rotblat B , Ansell K , Amelio I , Caraglia M , Misso G , Bernassola F , Cavasotto CN , Knight RA , Ciechanover A *et al* (2014) High throughput screening for inhibitors of the HECT ubiquitin E3 ligase ITCH identifies antidepressant drugs as regulators of autophagy. Cell Death Dis 5, e1203. 24787015 10.1038/cddis.2014.113 PMC4047876

[mol212567-bib-0075] Rotin D and Kumar S (2009) Physiological functions of the HECT family of ubiquitin ligases. Nat Rev Mol Cell Biol 10, 398–409. 19436320 10.1038/nrm2690

[mol212567-bib-0076] Sanarico AG , Ronchini C , Croce A , Memmi EM , Cammarata UA , De Antoni A , Lavorgna S , Divona M , Giacò L , Melloni GEM *et al* (2018) The E3 ubiquitin ligase WWP1 sustains the growth of acute myeloid leukaemia. Leukemia 32, 911–919. 29209041 10.1038/leu.2017.342 PMC5886071

[mol212567-bib-0077] Sane S , Hafner A , Srinivasan R , Masood D , Slunecka JL , Noldner CJ , Hanson AD , Kruisselbrink T , Wang X , Wang Y *et al* (2018) UBXN2A enhances CHIP‐mediated proteasomal degradation of oncoprotein mortalin‐2 in cancer cells. Mol Oncol 12, 1753–1777. 30107089 10.1002/1878-0261.12372 PMC6166003

[mol212567-bib-0078] Shi CS and Kehrl JH (2010) TRAF6 and A20 regulate lysine 63‐linked ubiquitination of Beclin‐1 to control TLR4‐induced autophagy. Sci Signal 3, ra42. 20501938 10.1126/scisignal.2000751 PMC6335036

[mol212567-bib-0079] Smith AG and Macleod KF (2019) Autophagy, cancer stem cells and drug resistance. J Pathol 247, 708–718. 30570140 10.1002/path.5222 PMC6668344

[mol212567-bib-0080] Staub O , Gautschi I , Ishikawa T , Breitschopf K , Ciechanover A , Schild L and Rotin D (1997) Regulation of stability and function of the epithelial Na+ channel (ENaC) by ubiquitination. EMBO J 16, 6325–6336. 9351815 10.1093/emboj/16.21.6325 PMC1170239

[mol212567-bib-0081] Strappazzon F , Nazio F , Corrado M , Cianfanelli V , Romagnoli A , Fimia GM , Campello S , Nardacci R , Piacentini M , Campanella M *et al* (2015) AMBRA1 is able to induce mitophagy via LC3 binding, regardless of PARKIN and p62/SQSTM1. Cell Death Differ 22, 419–432. 25215947 10.1038/cdd.2014.139 PMC4326570

[mol212567-bib-0082] Strasser A and Vaux DL (2018) Viewing BCL2 and cell death control from an evolutionary perspective. Cell Death Differ 25, 13–20. 29099481 10.1038/cdd.2017.145 PMC5729521

[mol212567-bib-0083] Sun A , Wei J , Childress C , Shaw JH 4th , Peng K , Shao G , Yang W and Lin Q (2017) The E3 ubiquitin ligase NEDD4 is an LC3‐interactive protein and regulates autophagy. Autophagy 13, 522–537. 28085563 10.1080/15548627.2016.1268301 PMC5361608

[mol212567-bib-0084] Sun Q , Zhang J , Fan W , Wong KN , Ding X , Chen S and Zhong Q (2011) The RUN domain of rubicon is important for hVps34 binding, lipid kinase inhibition, and autophagy suppression. J Biol Chem 286, 185–191. 21062745 10.1074/jbc.M110.126425 PMC3012973

[mol212567-bib-0085] Thompson JW , Nagel J , Hoving S , Gerrits B , Bauer A , Thomas JR , Kirschner MW , Schirle M and Luchansky SJ (2014) Quantitative Lys‐ε‐Gly‐Gly (diGly) proteomics coupled with inducible RNAi reveals ubiquitin‐mediated proteolysis of DNA damage‐inducible transcript 4 (DDIT4) by the E3 ligase HUWE1. J Biol Chem 289, 28942–28955. 25147182 10.1074/jbc.M114.573352 PMC4200252

[mol212567-bib-0086] Wan W , You Z , Zhou L , Xu Y , Peng C , Zhou T , Yi C , Shi Y and Liu W (2018) mTORC1‐regulated and HUWE1‐mediated WIPI2 degradation controls autophagy flux. Mol Cell 72, 303–315. 30340022 10.1016/j.molcel.2018.09.017

[mol212567-bib-0087] Wan L , Zou W , Gao D , Inuzuka H , Fukushima H , Berg AH , Drapp R , Shalk S , Hu D , Lester C *et al* (2011) Cdh1 regulates osteoblast function through an APC/C‐independent modulation of Smurf1. Mol Cell 44, 721–733. 22152476 10.1016/j.molcel.2011.09.024 PMC3240853

[mol212567-bib-0088] Wang H , Sun RQ , Camera D , Zeng XY , Jo E , Chan SM , Herbert TP , Molero JC and Ye JM (2016) Endoplasmic reticulum stress up‐regulates Nedd4‐2 to induce autophagy. FASEB J 30, 2549–2556. 27022162 10.1096/fj.201500119

[mol212567-bib-0089] Watt JE , Hughes GR , Walpole S , Monaco S , Stephenson GR , Bulman Page PC , Hemmings AM , Angulo J and Chantry A (2018) Discovery of Small Molecule WWP2 Ubiquitin Ligase Inhibitors. Chemistry 24, 17677–17680. 30207403 10.1002/chem.201804169

[mol212567-bib-0090] Xu P , Duong DM , Seyfried NT , Cheng D , Xie Y , Robert J , Rush J , Hochstrasser M , Finley D and Peng J (2009) Quantitative proteomics reveals the function of unconventional ubiquitin chains in proteasomal degradation. Cell 137, 133–145. 19345192 10.1016/j.cell.2009.01.041 PMC2668214

[mol212567-bib-0091] Yamano K , Matsuda N and Tanaka K (2016) The ubiquitin signal and autophagy: an orchestrated dance leading to mitochondrial degradation. EMBO Rep 17, 300–316. 26882551 10.15252/embr.201541486 PMC4772979

[mol212567-bib-0092] Yang Z and Klionsky DJ (2010) Mammalian autophagy: core molecular machinery and signaling regulation. Curr Opin Cell Biol 22, 124–131. 20034776 10.1016/j.ceb.2009.11.014 PMC2854249

[mol212567-bib-0093] Yorimitsu T and Klionsky DJ (2005) Autophagy: molecular machinery for self‐eating. Cell Death Differ 2, 1542–1552. 10.1038/sj.cdd.4401765 PMC1828868 16247502

[mol212567-bib-0094] Zhang L , Haraguchi S , Koda T , Hashimoto K and Nakagawara A (2011) Muscle atrophy and motor neuron degeneration in human NEDL1 transgenic mice. J Biomed Biotechnol 2011, 831092. 20976258 10.1155/2011/831092 PMC2952905

[mol212567-bib-0095] Zhong Q , Gao W , Du F and Wang X (2005) Mule/ARF‐BP1, a BH3‐only E3 ubiquitin ligase, catalyzes the polyubiquitination of Mcl‐1 and regulates apoptosis. Cell 121, 1085–1095. 15989957 10.1016/j.cell.2005.06.009

